# Supplementary figures and images for: Acetyl-carnitine improves hyperactivity and learning deficits in KAT6A haploinsufficient mice
Source: Life Sci Alliance. 2026 Feb 17;9(5):e202503549. doi: 10.26508/lsa.202503549 (PMC12912912; doi:10.26508/lsa.202503549)

# Figure 1A

E12 NSPCs

H3K23ac

pan H3 loading

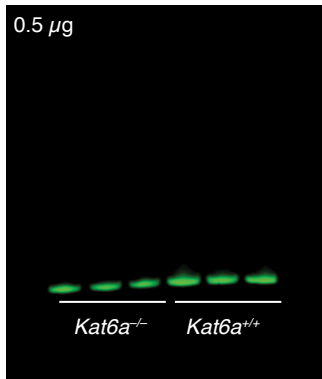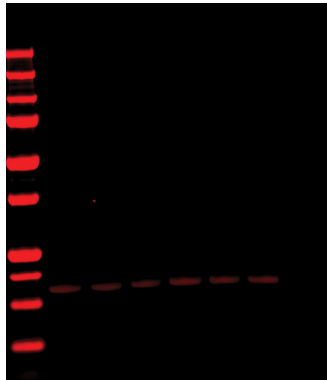

Supplement: Supplementary file 1 [file LSA-2025-03549_SdataF1.pdf]

# Figure S1A,C

E12 NSPCs

H3K9ac

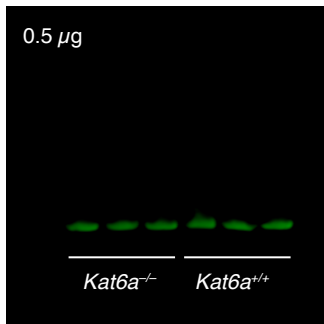

pan H3 loading

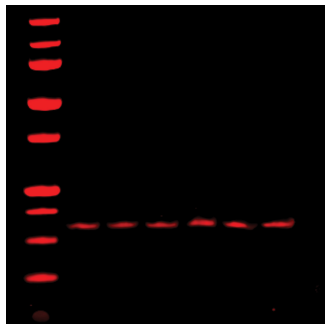

H3K14ac

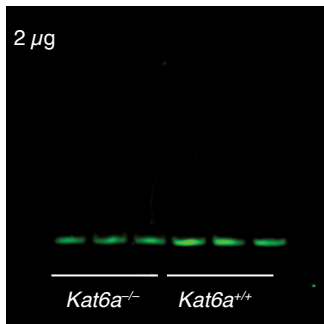

pan H3 loading

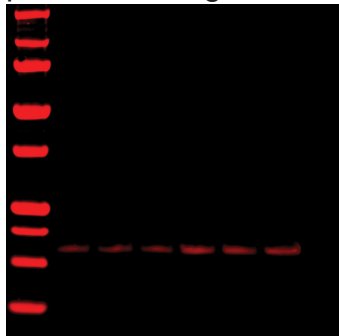

Supplement: Supplementary file 5 [file LSA-2025-03549_SdataFS1.pdf]
