# Supplementary material for: Acetyl-carnitine improves hyperactivity and learning deficits in KAT6A haploinsufficient mice
Source: Life Sci Alliance. 2026 Feb 17;9(5):e202503549. doi: 10.26508/lsa.202503549 (PMC12912912; doi:10.26508/lsa.202503549)
Supplement: Supplementary file 2 [file LSA-2025-03549_TableS1.docx]

**Table S1:** CRISPR/Cas9 crRNA, HDR and MiSeq primer sequences

| ***Protein sequence***  ***mutation*** | ***Coding sequence***  ***mutation*** | ***Reference*** | ***crRNA***  ***sequence*** | ***ssHDR sequence 5’ –> 3’*** | ***MiSeq Fdw primer***  ***with Overhang sequence 5’ –> 3’*** | ***MiSeq Rev primer***  ***with Overhang sequence 5’ –> 3’*** |
| --- | --- | --- | --- | --- | --- | --- |
| *Eco R1 Control* | N/A | N/A | CCTCTAA GGTTTGC TTACGA | AGCCATCTCTCTCCTTGC CAGAACCTCTAAGGTTTG CTTAGAATTCCGATGGAG CCAGAGAGGATCCTGGG AGGGAGAGCTTGGCA | GTGACCTATGAA CTCAGGAGTCCA GCTCAGGTTCTG GGAGAG | CTGAGACTTGCA CATCGCAGCCAT CCTTAGGCCTCC TCCTT |
| *p.R79** | c.235C>T |  | TTAGGAAGTGCTATTCGCCC | TCCAATTTTCCATGGTTCCGAGGCTTAGGAAGTGCTATTCAGCCAGGATTATCAGGATCTTTATAGGAATTGAGTCCTTTA | CAACTGCCCGCTTTATCAGT | AAGCAGAAACAGCGTCCTTC |
| *p.R269** | c.805C>T |  | ATTTTTGCCTTGATCTCGAC | TAGAAGGCAGAGATACTCACCGCATTTTTGCCTTGATCTCAGCAGGAGCTGCATGTTTTACACTCGATGCACTGCCACCGT | TGCTACTGGACCAAGTGTCTG | TTCATGAATGTTCTCCCTCGT |
| *p.E429Gfs*7* | c.1283_1284insT |  | CTGATGGGCGGAAAGCTCGG | TACCAAATTTTTTACCCCTTCCCCTGATGGGCGGAAAGCTCGGGGTGGAAGTGGTGGACTACTCTGAGCAATATCGAATCAGAAAG | TCTTACCTGTGGGCCAATCT | GGAAGGGGTAGGAAACGAAA |
| *p.D503Ifs*42* | c.1507delG |  | GACAGCGGACTTGTGGATCA | CTTCCCAAACTCAATGACAGAGGGACAGCGGACTTGTGGATAGGGGGACCAGTCACTCCAACTTTCTGTGCAGTCAAGAAA | CAAGATCCCTTCCAGTTCCA | TCCTTTGGAGTCCTGAGAAGA |
| *p.R1019** | c.3055C>T |  | TTCGGACTCTCCTCCTTCGG | ACTGCTATTGTGGTGTTTGCGCTTTCGGACTCTCCTCCTTCAATGGAGAAATGGTTTCTGTTTAATAGAGAAAGCAAGTATT | CGATCTCAAACGTGGGTTCT | TTTGGCAAACAACATCCTCA |
| *p.E1419Wfs*12* | c.4254_4257del (tgag) |  | AGAGATTCCTCATAGTGAGC | AGTTAATCGAATTAAAAGAGGAGGAAGAGATTCCTCATAGCTGGATCTGGAAACTGTACAGGCAGTGCAGTCTTTGACTC | TCGTCAGCCTGGGTGTAACT | AGCTGGATTCCGAAGAGGAG |
