# Supplementary material for: Acetyl-carnitine improves hyperactivity and learning deficits in KAT6A haploinsufficient mice
Source: Life Sci Alliance. 2026 Feb 17;9(5):e202503549. doi: 10.26508/lsa.202503549 (PMC12912912; doi:10.26508/lsa.202503549)
Supplement: Supplementary file 4 [file LSA-2025-03549_TableS3.docx]

**Table S3:** Single cell clone numbers of HEK293T cells with ARTHS mutations generated by CRISPR/Cas9 genome editing and clonal selection

| **ARTHS mutations** | **Single cell clone numbers for KAT6A mutations listed in Suppl. Table S2** | | | |
| --- | --- | --- | --- | --- |
| R79* | 10 | 20 | 22 |  |
| R269* | 9 | 11 | 31 | 36 |
| E429G | 3 | 9 | 12 | 13 |
| D503I | 4 | 12 | 13 | 29 |
| R1019* | 5 | 7 | 16 | 18 |
| E1419W | 2 | 9 | 21 | 34 |
