# Supplementary material for: Acetyl-carnitine improves hyperactivity and learning deficits in KAT6A haploinsufficient mice
Source: Life Sci Alliance. 2026 Feb 17;9(5):e202503549. doi: 10.26508/lsa.202503549 (PMC12912912; doi:10.26508/lsa.202503549)
Supplement: Supplementary file 9 [file LSA-2025-03549_TableS7.docx]

**Table S7:** Clinical features occurring in syndromes caused by mutations in genes downregulated (FDR < 0.05) in *Kat6a^–/–^* vs. *Kat6a^+/+^* dorsal telencephalon

| **Gene** | **Condition** | [**Inheritance**](https://research.nhgri.nih.gov/CGD/view/?par=general:gene,conditions:manifestation,intervention:All&l=Lgals4,Ppbp,Tbx1,Itgb3,Itga2b,Plek,Alx4,Rpl26,Rps25,Tomm5,Rpl22l1,Dct,Morf4l1,Vdac3,Rpl14,Tent5c,Itm2a,Apcdd1,Phgdh,Rnf7,Ltbp3,Dkk3,Ptma,Rpl23,Rps18,Oaz1,Nfatc4,Hnrnpa1,Slc4a1,Smarce1,Ajuba,Cacna1g,Derl2,Ccnb1,Tra2b,Hmgb2,Fzd1,Ube2n,Eif1,Mllt6,Hnrnpk,Adcy6,Npm1,Nfia,Tpm3,Isoc1,Ube2d3,Hmgn1,Sox3,Rps5,Nap1l1,Srsf3,Hnrnpa0,Rack1,Eif5a,Ddx17&n=1&g=ADCY6,ALX4,APCDD1,CACNA1G,DCT,EIF5A,HNRNPA1,HNRNPK,ITGA2B,ITGB3,LTBP3,NFIA,PHGDH,RPL26,SLC4A1,SMARCE1,SOX3,TBX1,TPM3)***** | [**Allelic conditions**](https://research.nhgri.nih.gov/CGD/view/?par=general:gene,conditions:manifestation,intervention:All&l=Lgals4,Ppbp,Tbx1,Itgb3,Itga2b,Plek,Alx4,Rpl26,Rps25,Tomm5,Rpl22l1,Dct,Morf4l1,Vdac3,Rpl14,Tent5c,Itm2a,Apcdd1,Phgdh,Rnf7,Ltbp3,Dkk3,Ptma,Rpl23,Rps18,Oaz1,Nfatc4,Hnrnpa1,Slc4a1,Smarce1,Ajuba,Cacna1g,Derl2,Ccnb1,Tra2b,Hmgb2,Fzd1,Ube2n,Eif1,Mllt6,Hnrnpk,Adcy6,Npm1,Nfia,Tpm3,Isoc1,Ube2d3,Hmgn1,Sox3,Rps5,Nap1l1,Srsf3,Hnrnpa0,Rack1,Eif5a,Ddx17&n=1&g=ADCY6,ALX4,APCDD1,CACNA1G,DCT,EIF5A,HNRNPA1,HNRNPK,ITGA2B,ITGB3,LTBP3,NFIA,PHGDH,RPL26,SLC4A1,SMARCE1,SOX3,TBX1,TPM3) | [**Manifestation categories**](https://research.nhgri.nih.gov/CGD/view/?par=general:gene,conditions:manifestation,intervention:All&l=Lgals4,Ppbp,Tbx1,Itgb3,Itga2b,Plek,Alx4,Rpl26,Rps25,Tomm5,Rpl22l1,Dct,Morf4l1,Vdac3,Rpl14,Tent5c,Itm2a,Apcdd1,Phgdh,Rnf7,Ltbp3,Dkk3,Ptma,Rpl23,Rps18,Oaz1,Nfatc4,Hnrnpa1,Slc4a1,Smarce1,Ajuba,Cacna1g,Derl2,Ccnb1,Tra2b,Hmgb2,Fzd1,Ube2n,Eif1,Mllt6,Hnrnpk,Adcy6,Npm1,Nfia,Tpm3,Isoc1,Ube2d3,Hmgn1,Sox3,Rps5,Nap1l1,Srsf3,Hnrnpa0,Rack1,Eif5a,Ddx17&n=1&g=ADCY6,ALX4,APCDD1,CACNA1G,DCT,EIF5A,HNRNPA1,HNRNPK,ITGA2B,ITGB3,LTBP3,NFIA,PHGDH,RPL26,SLC4A1,SMARCE1,SOX3,TBX1,TPM3) |
| --- | --- | --- | --- | --- |
| *ADCY6* | Lethal congenital contracture syndrome 8 | AR | [N/A](https://research.nhgri.nih.gov/CGD/view/?par=general:gene,conditions:manifestation,intervention:All&l=Lgals4,Ppbp,Tbx1,Itgb3,Itga2b,Plek,Alx4,Rpl26,Rps25,Tomm5,Rpl22l1,Dct,Morf4l1,Vdac3,Rpl14,Tent5c,Itm2a,Apcdd1,Phgdh,Rnf7,Ltbp3,Dkk3,Ptma,Rpl23,Rps18,Oaz1,Nfatc4,Hnrnpa1,Slc4a1,Smarce1,Ajuba,Cacna1g,Derl2,Ccnb1,Tra2b,Hmgb2,Fzd1,Ube2n,Eif1,Mllt6,Hnrnpk,Adcy6,Npm1,Nfia,Tpm3,Isoc1,Ube2d3,Hmgn1,Sox3,Rps5,Nap1l1,Srsf3,Hnrnpa0,Rack1,Eif5a,Ddx17&n=1&g=ADCY6,ALX4,APCDD1,CACNA1G,DCT,EIF5A,HNRNPA1,HNRNPK,ITGA2B,ITGB3,LTBP3,NFIA,PHGDH,RPL26,SLC4A1,SMARCE1,SOX3,TBX1,TPM3) | Musculoskeletal; Neurologic |
| *ALX4* | Parietal foramina 2; Frontonasal dysplasia 2  Variants may contribute to non-syndromic craniosynostosis | AD/AR | [N/A](https://research.nhgri.nih.gov/CGD/view/?par=general:gene,conditions:manifestation,intervention:All&l=Lgals4,Ppbp,Tbx1,Itgb3,Itga2b,Plek,Alx4,Rpl26,Rps25,Tomm5,Rpl22l1,Dct,Morf4l1,Vdac3,Rpl14,Tent5c,Itm2a,Apcdd1,Phgdh,Rnf7,Ltbp3,Dkk3,Ptma,Rpl23,Rps18,Oaz1,Nfatc4,Hnrnpa1,Slc4a1,Smarce1,Ajuba,Cacna1g,Derl2,Ccnb1,Tra2b,Hmgb2,Fzd1,Ube2n,Eif1,Mllt6,Hnrnpk,Adcy6,Npm1,Nfia,Tpm3,Isoc1,Ube2d3,Hmgn1,Sox3,Rps5,Nap1l1,Srsf3,Hnrnpa0,Rack1,Eif5a,Ddx17&n=1&g=ADCY6,ALX4,APCDD1,CACNA1G,DCT,EIF5A,HNRNPA1,HNRNPK,ITGA2B,ITGB3,LTBP3,NFIA,PHGDH,RPL26,SLC4A1,SMARCE1,SOX3,TBX1,TPM3) | Craniofacial; Dermatologic; Musculoskeletal; Neurologic |
| *APCDD1* | Hypotrichosis 1 | AD | [N/A](https://research.nhgri.nih.gov/CGD/view/?par=general:gene,conditions:manifestation,intervention:All&l=Lgals4,Ppbp,Tbx1,Itgb3,Itga2b,Plek,Alx4,Rpl26,Rps25,Tomm5,Rpl22l1,Dct,Morf4l1,Vdac3,Rpl14,Tent5c,Itm2a,Apcdd1,Phgdh,Rnf7,Ltbp3,Dkk3,Ptma,Rpl23,Rps18,Oaz1,Nfatc4,Hnrnpa1,Slc4a1,Smarce1,Ajuba,Cacna1g,Derl2,Ccnb1,Tra2b,Hmgb2,Fzd1,Ube2n,Eif1,Mllt6,Hnrnpk,Adcy6,Npm1,Nfia,Tpm3,Isoc1,Ube2d3,Hmgn1,Sox3,Rps5,Nap1l1,Srsf3,Hnrnpa0,Rack1,Eif5a,Ddx17&n=1&g=ADCY6,ALX4,APCDD1,CACNA1G,DCT,EIF5A,HNRNPA1,HNRNPK,ITGA2B,ITGB3,LTBP3,NFIA,PHGDH,RPL26,SLC4A1,SMARCE1,SOX3,TBX1,TPM3) | Dermatologic |
| *CACNA1G* | Spinocerebellar ataxia 42; Spinocerebellar ataxia 42, early-onset, severe, with neurodevelopmental deficits | AD | [N/A](https://research.nhgri.nih.gov/CGD/view/?par=general:gene,conditions:manifestation,intervention:All&l=Lgals4,Ppbp,Tbx1,Itgb3,Itga2b,Plek,Alx4,Rpl26,Rps25,Tomm5,Rpl22l1,Dct,Morf4l1,Vdac3,Rpl14,Tent5c,Itm2a,Apcdd1,Phgdh,Rnf7,Ltbp3,Dkk3,Ptma,Rpl23,Rps18,Oaz1,Nfatc4,Hnrnpa1,Slc4a1,Smarce1,Ajuba,Cacna1g,Derl2,Ccnb1,Tra2b,Hmgb2,Fzd1,Ube2n,Eif1,Mllt6,Hnrnpk,Adcy6,Npm1,Nfia,Tpm3,Isoc1,Ube2d3,Hmgn1,Sox3,Rps5,Nap1l1,Srsf3,Hnrnpa0,Rack1,Eif5a,Ddx17&n=1&g=ADCY6,ALX4,APCDD1,CACNA1G,DCT,EIF5A,HNRNPA1,HNRNPK,ITGA2B,ITGB3,LTBP3,NFIA,PHGDH,RPL26,SLC4A1,SMARCE1,SOX3,TBX1,TPM3) | Craniofacial; Musculoskeletal; Neurologic |
| *DCT* | Oculocutaneous albinism, type VIII | AR | [N/A](https://research.nhgri.nih.gov/CGD/view/?par=general:gene,conditions:manifestation,intervention:All&l=Lgals4,Ppbp,Tbx1,Itgb3,Itga2b,Plek,Alx4,Rpl26,Rps25,Tomm5,Rpl22l1,Dct,Morf4l1,Vdac3,Rpl14,Tent5c,Itm2a,Apcdd1,Phgdh,Rnf7,Ltbp3,Dkk3,Ptma,Rpl23,Rps18,Oaz1,Nfatc4,Hnrnpa1,Slc4a1,Smarce1,Ajuba,Cacna1g,Derl2,Ccnb1,Tra2b,Hmgb2,Fzd1,Ube2n,Eif1,Mllt6,Hnrnpk,Adcy6,Npm1,Nfia,Tpm3,Isoc1,Ube2d3,Hmgn1,Sox3,Rps5,Nap1l1,Srsf3,Hnrnpa0,Rack1,Eif5a,Ddx17&n=1&g=ADCY6,ALX4,APCDD1,CACNA1G,DCT,EIF5A,HNRNPA1,HNRNPK,ITGA2B,ITGB3,LTBP3,NFIA,PHGDH,RPL26,SLC4A1,SMARCE1,SOX3,TBX1,TPM3) | Dermatologic; Ophthalmologic |
| *EIF5A* | Faundes-Banka syndrome | AD |  | Cardiovascular; Craniofacial; Neurologic |
| *HNRNPA1* | Amyotrophic lateral sclerosis 20; Inclusion body myopathy with early-onset Paget disease with or without frontotemporal dementia 3; Myopathy, distal, 3 | AD | [N/A](https://research.nhgri.nih.gov/CGD/view/?par=general:gene,conditions:manifestation,intervention:All&l=Lgals4,Ppbp,Tbx1,Itgb3,Itga2b,Plek,Alx4,Rpl26,Rps25,Tomm5,Rpl22l1,Dct,Morf4l1,Vdac3,Rpl14,Tent5c,Itm2a,Apcdd1,Phgdh,Rnf7,Ltbp3,Dkk3,Ptma,Rpl23,Rps18,Oaz1,Nfatc4,Hnrnpa1,Slc4a1,Smarce1,Ajuba,Cacna1g,Derl2,Ccnb1,Tra2b,Hmgb2,Fzd1,Ube2n,Eif1,Mllt6,Hnrnpk,Adcy6,Npm1,Nfia,Tpm3,Isoc1,Ube2d3,Hmgn1,Sox3,Rps5,Nap1l1,Srsf3,Hnrnpa0,Rack1,Eif5a,Ddx17&n=1&g=ADCY6,ALX4,APCDD1,CACNA1G,DCT,EIF5A,HNRNPA1,HNRNPK,ITGA2B,ITGB3,LTBP3,NFIA,PHGDH,RPL26,SLC4A1,SMARCE1,SOX3,TBX1,TPM3) | Musculoskeletal; Neurologic |
| *HNRNPK* | Au-Kline syndrome | AD |  | Cardiovascular; Craniofacial; Musculoskeletal; Neurologic |
| *ITGA2B* | Bleeding disorder, platelet-type, 16, autosomal dominant; Glanzmann thrombasthenia 1 | AD/AR |  | Hematologic |
| *ITGB3* | Bleeding disorder, platelet-type, 24, autosomal dominant; Glanzmann thrombasthenia 2; Thrombocytopenia, neonatal alloimmune  Bi-allelic variants have been described as resulting in a more severe phenotype; Specific variants may also be important in neonatal alloimune thrombocytopenia (as relates to platelet transfusions) | AD/AR |  | Hematologic |
| *LTBP3* | Geleophysic dysplasia 3; Dental anomalies and short stature | AD/AR | [N/A](https://research.nhgri.nih.gov/CGD/view/?par=general:gene,conditions:manifestation,intervention:All&l=Lgals4,Ppbp,Tbx1,Itgb3,Itga2b,Plek,Alx4,Rpl26,Rps25,Tomm5,Rpl22l1,Dct,Morf4l1,Vdac3,Rpl14,Tent5c,Itm2a,Apcdd1,Phgdh,Rnf7,Ltbp3,Dkk3,Ptma,Rpl23,Rps18,Oaz1,Nfatc4,Hnrnpa1,Slc4a1,Smarce1,Ajuba,Cacna1g,Derl2,Ccnb1,Tra2b,Hmgb2,Fzd1,Ube2n,Eif1,Mllt6,Hnrnpk,Adcy6,Npm1,Nfia,Tpm3,Isoc1,Ube2d3,Hmgn1,Sox3,Rps5,Nap1l1,Srsf3,Hnrnpa0,Rack1,Eif5a,Ddx17&n=1&g=ADCY6,ALX4,APCDD1,CACNA1G,DCT,EIF5A,HNRNPA1,HNRNPK,ITGA2B,ITGB3,LTBP3,NFIA,PHGDH,RPL26,SLC4A1,SMARCE1,SOX3,TBX1,TPM3) | Craniofacial; Dental; Musculoskeletal |
| *NFIA* | Brain malformations with or without urinary tract defects | AD |  | Neurologic; Renal |
| *PHGDH* | Phosphoglycerate dehydrogenase deficiency | AR | Allelic with Neu-Laxova syndrome 1 (AR) | Biochemical; Craniofacial; Dermatologic; Endocrine; Musculoskeletal; Neurologic; Ophthalmologic; Pulmonary |
| *RPL26* | Diamond-Blackfan anemia 11 | AD |  | Audiologic/Otolaryngologic; Cardiovascular; Craniofacial; Hematologic; Musculoskeletal; Oncologic; Renal |
| *SLC4A1* | Spherocytosis, type 4; Ovalcytosis, Southeast Asian; Cryohydrocytosis; Renal tubular acidosis, distal, with hemolytic anemia; Renal tubular acidosis, distal, 1; Renal tubular acidosis, distal, autosomal recessive; Blood group, Wright; Blood group, Waldner; Blood group, Diego; Blood group, Froese; Blood group, Swann | AD/AR/BG | Allelic with Acanthocytosis, Band 3 Memphis (AR) | Hematologic; Renal |
| *SMARCE1* | Meningioma, familial, susceptibility to | AD | Allelic with Coffin-Siris syndrome 5 (AD) | Craniofacial; Musculoskeletal; Neurologic; Oncologic |
| *SOX3* | Intellectual developmental disorder, X-linked, with panyhypopituitarism; Panhypopituitarism, X-linked  Deletions/insertions involving SOX3 regulatory regions can result in 46,XX sex reversal 3 (XL) or Hypoparathyroidism, X-linked by a position effect (XL) | XL |  | Endocrine; Musculoskeletal; Neurologic |
| *TBX1* | Conotruncal anomaly face syndrome; Tetralogy of Fallot | AD |  | Cardiovascular; Craniofacial; Neurologic |
| *TPM3* | Congenital myopathy 4A, autosomal dominant; Congenital myopathy 4B, autosomal recessive | AD/AR | [N/A](https://research.nhgri.nih.gov/CGD/view/?par=general:gene,conditions:manifestation,intervention:All&l=Lgals4,Ppbp,Tbx1,Itgb3,Itga2b,Plek,Alx4,Rpl26,Rps25,Tomm5,Rpl22l1,Dct,Morf4l1,Vdac3,Rpl14,Tent5c,Itm2a,Apcdd1,Phgdh,Rnf7,Ltbp3,Dkk3,Ptma,Rpl23,Rps18,Oaz1,Nfatc4,Hnrnpa1,Slc4a1,Smarce1,Ajuba,Cacna1g,Derl2,Ccnb1,Tra2b,Hmgb2,Fzd1,Ube2n,Eif1,Mllt6,Hnrnpk,Adcy6,Npm1,Nfia,Tpm3,Isoc1,Ube2d3,Hmgn1,Sox3,Rps5,Nap1l1,Srsf3,Hnrnpa0,Rack1,Eif5a,Ddx17&n=1&g=ADCY6,ALX4,APCDD1,CACNA1G,DCT,EIF5A,HNRNPA1,HNRNPK,ITGA2B,ITGB3,LTBP3,NFIA,PHGDH,RPL26,SLC4A1,SMARCE1,SOX3,TBX1,TPM3) | Craniofacial; Musculoskeletal |

*AD, autosomal dominant; AR, autosomal recessive; XL, X-linked.
