# Supplementary material for: Acetyl-carnitine improves hyperactivity and learning deficits in KAT6A haploinsufficient mice
Source: Life Sci Alliance. 2026 Feb 17;9(5):e202503549. doi: 10.26508/lsa.202503549 (PMC12912912; doi:10.26508/lsa.202503549)
Supplement: Supplementary file 10 [file LSA-2025-03549_TableS8.docx]

**Table S8:** Clinical features occurring in syndromes caused by mutations in genes downregulated (FDR < 0.05) in *Kat6a^+/–^* vs. *Kat6a^+/+^* dorsal telencephalon

| **Gene** | **Condition** | [**Inheritance**](https://research.nhgri.nih.gov/CGD/view/?par=general:gene,conditions:manifestation,intervention:All&l=Pf4,Ppp1r14c,Rps16,Rps25,Tomm7,Cenpw,Rpl26,Pfdn5,Rpl14,Serf1,Atp6v0c,Snrpd2,H3f3a,Morf4l1,Eid2,Basp1,Ppp1r14b,Ndufa13,H1f10,Tmsb4x,Rnf7,Fezf2,Scrt2,Igfbpl1,Tpm3,Ctxn1,Eif1,Rpl22,Odc1,Plppr3,Npm1,Hnrnpa1,Hnrnpk,Calm2,Hmgn1,Ddx5,Hnrnpa0,Rpl8&n=1&g=ATP6V0C,CALM2,HNRNPA1,HNRNPK,NDUFA13,ODC1,RPL26,TOMM7,TPM3)***** | [**Allelic conditions**](https://research.nhgri.nih.gov/CGD/view/?par=general:gene,conditions:manifestation,intervention:All&l=Pf4,Ppp1r14c,Rps16,Rps25,Tomm7,Cenpw,Rpl26,Pfdn5,Rpl14,Serf1,Atp6v0c,Snrpd2,H3f3a,Morf4l1,Eid2,Basp1,Ppp1r14b,Ndufa13,H1f10,Tmsb4x,Rnf7,Fezf2,Scrt2,Igfbpl1,Tpm3,Ctxn1,Eif1,Rpl22,Odc1,Plppr3,Npm1,Hnrnpa1,Hnrnpk,Calm2,Hmgn1,Ddx5,Hnrnpa0,Rpl8&n=1&g=ATP6V0C,CALM2,HNRNPA1,HNRNPK,NDUFA13,ODC1,RPL26,TOMM7,TPM3) | [**Manifestation categories**](https://research.nhgri.nih.gov/CGD/view/?par=general:gene,conditions:manifestation,intervention:All&l=Pf4,Ppp1r14c,Rps16,Rps25,Tomm7,Cenpw,Rpl26,Pfdn5,Rpl14,Serf1,Atp6v0c,Snrpd2,H3f3a,Morf4l1,Eid2,Basp1,Ppp1r14b,Ndufa13,H1f10,Tmsb4x,Rnf7,Fezf2,Scrt2,Igfbpl1,Tpm3,Ctxn1,Eif1,Rpl22,Odc1,Plppr3,Npm1,Hnrnpa1,Hnrnpk,Calm2,Hmgn1,Ddx5,Hnrnpa0,Rpl8&n=1&g=ATP6V0C,CALM2,HNRNPA1,HNRNPK,NDUFA13,ODC1,RPL26,TOMM7,TPM3) |
| --- | --- | --- | --- | --- |
| *ATP6V0C* | Epilepsy, early onset 3, with or without developmental delay | AD | [N/A](https://research.nhgri.nih.gov/CGD/view/?par=general:gene,conditions:manifestation,intervention:All&l=Pf4,Ppp1r14c,Rps16,Rps25,Tomm7,Cenpw,Rpl26,Pfdn5,Rpl14,Serf1,Atp6v0c,Snrpd2,H3f3a,Morf4l1,Eid2,Basp1,Ppp1r14b,Ndufa13,H1f10,Tmsb4x,Rnf7,Fezf2,Scrt2,Igfbpl1,Tpm3,Ctxn1,Eif1,Rpl22,Odc1,Plppr3,Npm1,Hnrnpa1,Hnrnpk,Calm2,Hmgn1,Ddx5,Hnrnpa0,Rpl8&n=1&g=ATP6V0C,CALM2,HNRNPA1,HNRNPK,NDUFA13,ODC1,RPL26,TOMM7,TPM3) | Cardiovascular; Craniofacial; Dental; Neurologic |
| *CALM2* | Long QT syndrome 15 | AD |  | Cardiovascular; Neurologic |
| *HNRNPA1* | Amyotrophic lateral sclerosis 20; Inclusion body myopathy with early-onset Paget disease with or without frontotemporal dementia 3; Myopathy, distal, 3 | AD | [N/A](https://research.nhgri.nih.gov/CGD/view/?par=general:gene,conditions:manifestation,intervention:All&l=Pf4,Ppp1r14c,Rps16,Rps25,Tomm7,Cenpw,Rpl26,Pfdn5,Rpl14,Serf1,Atp6v0c,Snrpd2,H3f3a,Morf4l1,Eid2,Basp1,Ppp1r14b,Ndufa13,H1f10,Tmsb4x,Rnf7,Fezf2,Scrt2,Igfbpl1,Tpm3,Ctxn1,Eif1,Rpl22,Odc1,Plppr3,Npm1,Hnrnpa1,Hnrnpk,Calm2,Hmgn1,Ddx5,Hnrnpa0,Rpl8&n=1&g=ATP6V0C,CALM2,HNRNPA1,HNRNPK,NDUFA13,ODC1,RPL26,TOMM7,TPM3) | Musculoskeletal; Neurologic |
| *HNRNPK* | Au-Kline syndrome | AD |  | Cardiovascular; Craniofacial; Musculoskeletal; Neurologic |
| *NDUFA13* | Thyroid carcinoma, Hurthle cell | AD | Allelic with Mitochondrial complex I deficiency, nuclear type 28 (AR) | Biochemical; Neurologic; Oncologic; Ophthalmologic |
| *ODC1* | Neurodevelopmental disorder with alopecia and brain imaging abnormalities (Bachmann-Bupp syndrome) | AD | [N/A](https://research.nhgri.nih.gov/CGD/view/?par=general:gene,conditions:manifestation,intervention:All&l=Pf4,Ppp1r14c,Rps16,Rps25,Tomm7,Cenpw,Rpl26,Pfdn5,Rpl14,Serf1,Atp6v0c,Snrpd2,H3f3a,Morf4l1,Eid2,Basp1,Ppp1r14b,Ndufa13,H1f10,Tmsb4x,Rnf7,Fezf2,Scrt2,Igfbpl1,Tpm3,Ctxn1,Eif1,Rpl22,Odc1,Plppr3,Npm1,Hnrnpa1,Hnrnpk,Calm2,Hmgn1,Ddx5,Hnrnpa0,Rpl8&n=1&g=ATP6V0C,CALM2,HNRNPA1,HNRNPK,NDUFA13,ODC1,RPL26,TOMM7,TPM3) | Craniofacial; Dermatologic; Musculoskeletal; Neurologic |
| *RPL26* | Diamond-Blackfan anemia 11 | AD |  | Audiologic/Otolaryngologic; Cardiovascular; Craniofacial; Hematologic; Musculoskeletal; Oncologic; Renal |
| *TOMM7* | Garg-Mishra progeroid syndrome | AR | [N/A](https://research.nhgri.nih.gov/CGD/view/?par=general:gene,conditions:manifestation,intervention:All&l=Pf4,Ppp1r14c,Rps16,Rps25,Tomm7,Cenpw,Rpl26,Pfdn5,Rpl14,Serf1,Atp6v0c,Snrpd2,H3f3a,Morf4l1,Eid2,Basp1,Ppp1r14b,Ndufa13,H1f10,Tmsb4x,Rnf7,Fezf2,Scrt2,Igfbpl1,Tpm3,Ctxn1,Eif1,Rpl22,Odc1,Plppr3,Npm1,Hnrnpa1,Hnrnpk,Calm2,Hmgn1,Ddx5,Hnrnpa0,Rpl8&n=1&g=ATP6V0C,CALM2,HNRNPA1,HNRNPK,NDUFA13,ODC1,RPL26,TOMM7,TPM3) | Craniofacial; Musculoskeletal; Neurologic |
| *TPM3* | Congenital myopathy 4A, autosomal dominant; Congenital myopathy 4B, autosomal recessive | AD/AR | [N/A](https://research.nhgri.nih.gov/CGD/view/?par=general:gene,conditions:manifestation,intervention:All&l=Pf4,Ppp1r14c,Rps16,Rps25,Tomm7,Cenpw,Rpl26,Pfdn5,Rpl14,Serf1,Atp6v0c,Snrpd2,H3f3a,Morf4l1,Eid2,Basp1,Ppp1r14b,Ndufa13,H1f10,Tmsb4x,Rnf7,Fezf2,Scrt2,Igfbpl1,Tpm3,Ctxn1,Eif1,Rpl22,Odc1,Plppr3,Npm1,Hnrnpa1,Hnrnpk,Calm2,Hmgn1,Ddx5,Hnrnpa0,Rpl8&n=1&g=ATP6V0C,CALM2,HNRNPA1,HNRNPK,NDUFA13,ODC1,RPL26,TOMM7,TPM3) | Craniofacial; Musculoskeletal |

*AD, autosomal dominant; AR, autosomal recessive; XL, X-linked.
