# Supplementary material for: Acetyl-carnitine improves hyperactivity and learning deficits in KAT6A haploinsufficient mice
Source: Life Sci Alliance. 2026 Feb 17;9(5):e202503549. doi: 10.26508/lsa.202503549 (PMC12912912; doi:10.26508/lsa.202503549)
Supplement: Supplementary file 11 [file LSA-2025-03549_TableS9.docx]

**Table S9:** RT-qPCR primers

| *Detected mRNA* | *Primer sequence* | *Reference* |
| --- | --- | --- |
| *Kat6a (human)* | F 5’-CTGACTCCGAGAGGCCAATG-3’  R 5’-TGGATTGGTTTGCGGCTCTT-3’ | This study |
| *GAPDH (human)* | F 5’-TGCACCACCAACTGCTTAGC-3’  R 5’-GGCATGGACTGTGGTCATGAG-3’ | Wichmann et al., 2022 (1) |
| *HSP90AB1* (human) | F 5’-CACCTTGCTGCATTGTGACC-3’  R 5’-AGGAACTGCAGCATTGGGTT-3’ | This study |
