# Supplementary material for: Acetyl-carnitine improves hyperactivity and learning deficits in KAT6A haploinsufficient mice
Source: Life Sci Alliance. 2026 Feb 17;9(5):e202503549. doi: 10.26508/lsa.202503549 (PMC12912912; doi:10.26508/lsa.202503549)
Supplement: Supplementary file 12 [file LSA-2025-03549_TableS10.docx]

**Table S10:** Genotyping primers for three-way PCR

| *Detected allele* | *Primer sequence* | *PCR amplicon* |
| --- | --- | --- |
| *Kat6a^+^* unique | R 5’-AGAAGTACAGTGCTTTGGTTTCC-3’ | 358 bp |
| *Kat6a^–^* unique | R 5’-ATAGGAACTTCATCAGTCAGGTAC-3’ | 172 bp |
| Common primer | F 5’-TTCTTGACCTCTGTGTCGTGTGC-3’ |  |
