# Supplementary material for: Acetyl-carnitine improves hyperactivity and learning deficits in KAT6A haploinsufficient mice
Source: Life Sci Alliance. 2026 Feb 17;9(5):e202503549. doi: 10.26508/lsa.202503549 (PMC12912912; doi:10.26508/lsa.202503549)
Supplement: Supplementary file 13 [file LSA-2025-03549_TableS11.docx]

**Table S11:** Buffers and media

| ***Buffer*** | ***Recipe*** |
| --- | --- |
| *2% FACS buffer pH 7.1 - 7.2* | 2% (vol/vol) FCS, 150 mM NaCl, 3.7 mM KCl, 2.5 mM CaCl2•2H2O, 1.2 mM MgSO4•7H2O, 0.8 mM K2HPO4, 1.2 mM KH2PO4, 11.5 mM HEPES, made up in MQ-H2O |
| *PBS* | 16 mM Na2HPO42•H2O, 0.4 mM NaH2PO4•H2O, 150 mM NaCl, in MQ- H2O, pH 7.4 |
| *Red cell lysis buffer pH 7.2* | 150 mM ammonium chloride (NH4Cl), 0.1 mM EDTA, 12 mM NaHCO3 made up in MQ-H2O |
| *Nuclei extraction buffer (NIB)* | 15 mM Tris pH 7.5, 60 mM KCl, 15 mM NaCl, 5 mM MgCl_2_, 1 mM CaCl_2_, 250 mM sucrose, protease inhibitors (EDTA-free tablets), sodium butyrate, PMSF)  with 0.2% NP-40 alternative buffer |
| *Foetal cortical neuron culture medium* | Neurobasal medium (Gibco, 21103049), 2% B-27 (Gibco, 17504044), 1% GlutaMAX, 1% penicillin/streptomycin |
| *Post-natal cortical neuron HAG medium* | Hibernate-A medium (Gibco, A1247501), 1% GlutaMAX (Gibco, 35050061) |
| *BSA solution* | 0.5% BSA (Sigma-Aldrich, A8806) in PBS |
| *Post-natal cortical neuron culture medium* | Neurobasal-A medium (Gibco, 10888022), 2% B-27 plus (Gibco, A3582801), 1% GlutaMAX,10 ug/mL Gentamicin (Gibco, 15710064) |
| *2x SDS sample buffer* | 125mM Tris HCl, pH 6.8, 20% Glycerol, 4% SDS, 0.005% Bromophenol Blue, 0.1M DTT |
