# Supplementary material for: Acetyl-carnitine improves hyperactivity and learning deficits in KAT6A haploinsufficient mice
Source: Life Sci Alliance. 2026 Feb 17;9(5):e202503549. doi: 10.26508/lsa.202503549 (PMC12912912; doi:10.26508/lsa.202503549)
Supplement: Supplementary file 14 [file LSA-2025-03549_TableS12.docx]

**Table S12:** Antibodies

| ***Antigen*** | ***Fluorophore*** | ***Clone number/order number*** | ***Supplier*** |
| --- | --- | --- | --- |
| *B220* | A700 | B220 Clone 16A | Made in house |
| *CD19* | PECy7 | Clone 1D3/ #552854 | BD Pharmingen |
| *CD4* | APC | GK1.5/#553730 | BD Pharmingen |
| *CD8* | PE | Clone 56.3.7 | Made in house |
| *GR1* | A594 | Clone 1A8 | Made in house |
| *MAC1/LYG6* | PerCP/CY5.5 | Clone ICRF44/#301327 | BioLegend |
| *H3K9ac* | Unconjugated | C5B11/#9649 | Cell signalling |
| *H3K9ac* | Unconjugated | Monoclonal/#13-0033 | Epicypher |
| *H3K14ac* | Unconjugated | Cone EP964Y/#Ab52946 | Abcam |
| *H3K14ac* | Unconjugated | Polyclonal/07-353 | Millipore |
| *H3K23ac* | Unconjugated | Polyclonal/07-355 | Millipore |
| *Pan H3* | Unconjugated | Sp2/0-Ag14/ ab10779 | Abcam |
| *Beta III-tubulin* | Unconjugated | Clone 5G8/ G7121 | Promega, |
| *LIVE/DEAD Fixable dead cell stain* | A488 | L23101 | ThermoFisher |
| *Viability marker* | Flurogold | Polyclonal/AB153-I | Sigma-Aldrich |
| *Anti-rabbit secondary for FACS* | Pacific Blue | Polyclonal/111-475-003 | Jackson Immunoresearch |
| *Anti-rabbit secondary for western immunoblotting* | IRDye® 680 | Polyclonal/926-68071 | Li-Cor |
| *Anti-Mouse secondary for western immunoblotting* | IRDye® 800 | Polyclonal/926-32210 | Li-Cor |
| *Anti-mouse IgG1 secondary for immunoflorescence* | *AlexaFluor 568* | Polyclonal/ A-21124 | Invitrogen |
